# Supplementary material for: ProteinShader: illustrative rendering of macromolecules
Source: BMC Struct Biol. 2009 Mar 30;9:19. doi: 10.1186/1472-6807-9-19 (PMC2672931; doi:10.1186/1472-6807-9-19)
Supplement: Additional file 1 — ProteinShader program without source code. This compressed file contains the complete ProteinShader program including associated libraries, but no source code. A README.txt file gives an overview of the ProteinShader distribution, and the index.html file in the help subdirectory has directions on getting started with the program as well as a set of tutorials. [file 1472-6807-9-19-S1.zip › ProteinShader-beta-0_9_4-binary/help/api/org/proteinshader/structure/visitor/Visitor.html]

Visitor (ProteinShader API)


|  |  |  |
| --- | --- | --- |
| **Overview** \| **Package** \| **Class** \| **Use** \| **Tree** \| **Deprecated** \| **Index** \| **Help** | |  |
| **PREV CLASS**   NEXT CLASS | **FRAMES**    **NO FRAMES**     **All Classes** |  |
| SUMMARY: NESTED \| FIELD \| CONSTR \| METHOD | DETAIL: FIELD \| CONSTR \| METHOD |  |


---


## org.proteinshader.structure.visitor Class Visitor

```
java.lang.Object
  org.proteinshader.structure.visitor.Visitor
```

**Direct Known Subclasses:**: AminoAcidLabelVisitor, BondDestroyerVisitor, BondGeneratorVisitor, BoundsVisitor, FrenetFrameGeneratorVisitor, LoopGeneratorVisitor, ModifierVisitor, SFWriterVisitor, VisibilityVisitor

---

``` public class Visitor extends Object ```

This self-propelled Visitor knows how to traverse the hierarchy of
data objects contained by a Structure.
